# DELIVERING HIGH-INTENSITY STRENGTH TRAINING AND MAINTAINING INTERVENTION FIDELITY: THE STEP-HI STUDY

**DOI:** 10.1093/geroni/igae098.0273

**Published:** 2024-12-31

**Authors:** Michelle Rauzi, Melissa Tran, Kelly Monroe, Jennifer Stevens-Lapsley

**Affiliations:** Department of Veterans Affairs, Aurora, Colorado, United States; University of Colorado Anschutz Medical Campus, Aurora, Colorado, United States; Washington University School of Medicine, St. Louis, Missouri, United States; University of Colorado Anschutz Medical Campus, Aurora, Colorado, United States

## Abstract

Hip fractures in women can result in significant functional decline. The STEP-HI study evaluated the effect of testosterone therapy versus placebo combined with supervised exercise training on functional recovery following hip fracture. Maximizing internal validity was essential to maintain rigor of this study. Further, knowledge of both the intervention protocol and the fidelity process are essential to understand threats to internal validity, identify barriers to the protocol, and implement appropriate solutions. Our purpose is to provide an overview of the supervised exercise training intervention and discuss the advantages of and barriers to a remote intervention fidelity monitoring process. Twenty-eight exercise interventionists (EI) across 8 sites delivered the supervised exercise training protocol. EI training was included during the initial step of a 3-step fidelity process: Step 1 Training Certification, Step 2 Initial Fidelity, and Step 3 Ongoing Fidelity. The fidelity process was managed from one site that assessed intervention fidelity through videos submitted approximately every 6 months by each site. Supervising physical therapists oversaw fidelity at their respective site and completed in-person fidelity assessments once per month. The primary advantage of this process was cost savings that occurred because there were no site visits with associated travel costs. A significant barrier was technological challenges related to recording and uploading videos to a secure file-sharing website. Across all sites, 140 fidelity assessments were completed; the mean (SD) score was 96.3% (3.4%) indicating excellent intervention fidelity. Our procedures and lessons learned will guide planning for future clinical trials of complex interventions.

